# Temporal Trends of Transcatheter Aortic Valve Implantation over 12 Years: A High-Volume Single-Center Experience

**DOI:** 10.3390/jcm11174962

**Published:** 2022-08-24

**Authors:** Shir Frydman, David Zahler, Ilan Merdler, Ophir Freund, Yacov Shacham, Shmuel Banai, Ariel Finkelstein, Arie Steinvil

**Affiliations:** Department of Cardiology, Tel-Aviv Sourasky Medical Center, Sackler Faculty of Medicine, Tel-Aviv University, Tel-Aviv 6997801, Israel

**Keywords:** TAVR, trends, complications, outcomes, mortality

## Abstract

Transcatheter aortic valve replacement (TAVR) has become the mainstay of treatment for patients with severe AS. Since the TAVR population and patients’ outcomes have dramatically changed over the last decade, updated data regarding contemporary practice and trends are pertinent to clinical use. We performed a retrospective observational analysis of consecutive patient who underwent TAVR for symptomatic severe AS between the years 2009 and 2021 in a single high-volume center. Patients were divided into four equal time groups based on the procedure date (2009–2012, 2013–2015, 2016–2018 and 2019–2021). A total of 1988 patients were included in this study and divided into four groups, with 321, 482, 565 and 620 patients in groups 1–4, respectively. Significant trends were seen in baseline characteristics of a few parameters, including lower age, lower procedural risk and reduced rates of comorbidity (*p* for trend < 0.0001 for all factors mentioned above). A shift was seen in the procedural technique with lower balloon pre-dilatation and higher device success rates (*p* for trend < 0.0001). The post-procedural period changed over the years with fewer pacemaker placements (*p* < 0.0001) and reduced rates of AKI and post-procedural bleed (*p* value 0.02 and <0.0001, respectively). Furthermore, overall hospital stay was shortened from 7 ± 7.1 days to 2.3 ± 1.7 (*p* < 0.0001). Finally, patient follow up revealed reduced mortality rates at 30 days (*p* < 0.0001) and 1 year (*p* = 0.013). Multivariate regression revealed that a late implantation date was an independent protector from mortality (HR 0.84, *p* = 0.002). In conclusion, our study demonstrated that TAVR has become a safer practice over the years with reduced rates of morbidity and mortality.

## 1. Introduction

Transcatheter aortic valve replacement (TAVR) was first introduced in 2002 and since then has been rapidly evolving. Once reserved only for patient with high surgical risk, it is now becoming available for a wider, more diverse patient population [1,2,3,4]. The European Society of Cardiology (ESC) guidelines for valvular disease recommend considering TAVR for all patients above 75 years of age, as well as for younger patients with unfavorable or high surgical risk, while the American Heart Association recently added a class IIa recommendation for intermediate surgical risk [5,6].

Yet, this procedure carries with it some well-known complications, including, among others, a permanent pacemaker (PPM), paravalvular leaks (PVLs) and bleeding and vascular or neurological complications [4,7]. These complications were described and published by the Valve Academic Research Consortium (VARC) [8,9]. The rate of adverse outcomes has varied over the years and was estimated to be as high as 33% in an early meta-analysis [10]. With newly developed valves, delivery methods and increasing operator experience, the safety profile of the procedure has improved, resulting in lower rates of peri-procedural complications, paravalvular regurgitation and mortality [11].

Several registries have provided real-life data regarding TAVR outcomes [12,13,14]; however, updated reports on temporal trends in large populations are scarce.

This study aimed to assess the temporal trends in the TAVR procedure in a series of consecutive patients from 2009 to 2021 in a large-scale tertiary center. We hypothesized that there would be a shift to a younger patient population as well as reduced rates of significant complications.

## 2. Materials and Methods

This was a retrospective, single-center observational analysis of consecutive patients who underwent TAVR for symptomatic severe AS at a university-affiliated tertiary referral center. All patients were considered intermediate- and high-risk for valvular surgery by our institutional heart team. We excluded 174 patients with nonstandard features; additional interventions; valve-in-valve, nonfemoral access concomitant coronary per cutaneous intervention (PCI); concomitant other procedure; and missing data. Included in the cohort were 1988 patients with severe aortic stenosis who underwent TAVR between the years 2009 and 2021 and were registered in the Tel-Aviv Sourasky medical center TAVR registry. All patients underwent clinical and echocardiographic evaluation as a baseline evaluation in our designated TAVR clinic. Clinical, echocardiographic and procedural variables were collected from the electronic medical records after patients signed consent. Outcomes and complications were defined by the Valve Academic Research Consortium (VARC-2) criteria [8]. Mortality data were abstracted from the medical center’s electronic medical records, which is automatically updated with the mortality data of the Israeli Ministry of the Interior affairs.

The study was approved by the Sourasky Medical Center review board (0409–11-TLV) and conducted in accordance with the Declaration of Helsinki.

### Statistical Methods

Continuous variables were presented as mean ± standard deviation when normally distributed. Median and interquartile ranges were used in cases of non-normally distributed continuous variables. Distribution was evaluated with Q-Q plots and a histogram. Categorical variables were presented as absolute number and percentages.

Linear trend in-between groups for categorical variables was evaluated with the Mantel–Haenszel Linear-by-Linear association test. Analysis of variance (ANOVA) with polynomial contrasts was used for normally distributed continuous variables. The Jonckheere trend test was used for non-normally distributed continuous variables.

One-year survival rates were described using the Kaplan–Meier method. Differences between study groups were assessed with the log-rank test.

The effects of clinical and echocardiographic variables were evaluated with multivariate cox proportional-hazards models including all baseline variables found to be significant in univariate analysis.

A two-tailed *p* value of <0.05 was considered significant for all analyses. All analyses were performed with the SPSS software (SPSS Inc., Version 23, Chicago, IL, USA).

## 3. Results

### 3.1. Baseline Characteristics

A total of 1988 patients were included in this cohort and divided into four groups based on the intervention date: years 2009–2012 (group 1), 2013–2015 (group 2), 2016–2018 (group 3) and 2019–2021 (group 4), including 321, 482, 565 and 620 patients, respectively. Baseline characteristics are demonstrated in Table 1. Over the study period, a significant trend was seen over the years in the following parameters: lower age (84 ± 5.6, 83.7 ± 6.2, 82.1 ± 6.3 and 80.3 ± 6.6 in groups 1–4, respectively *p* < 0.0001), lower female ratio (60%, 53%, 52% and 44% in groups 1–4, respectively *p* < 0.0001), lower body surface area (1.79 ± 0.21, 1.8 ± 0.19, 1.81 ± 0.21 and 1.85 ± 0.21, *p* < 0.0001), lower risk according to Euroscore2 (4.6 (3–7.3), 4.3 (2.6–7.4), 3.4 (2.2–5.6) and 2.8 (1.8–5.6) in group 1–4, respectively *p* < 0.0001), reduced rates of New York Heart Association functional class (NYHA) > 2 (97%, 86%, 85% and 74% in groups 1–4, respectively, *p* < 0.0001), lower rates of hypertension (88%, 87%, 84% and 71% in groups 1–4, respectively, *p* = 0.001), lower rates of chronic obstructive pulmonary disease (20%, 12%, 9% and 8% in groups 1–4, respectively, *p* = 0.001), lower rates of prior myocardial infraction (16%, 20%, 9% and 5% in groups 1–4, respectively, *p* < 0.0001) and coronary artery disease (57%, 52%, 43% and 47% in groups 1–4, respectively, *p* < 0.0001), lower baseline hemoglobin (11.9 ± 1.4, 11.9 ± 1.5, 12.1 ± 1.6 and 12.6 ± 1.6 in groups 1–4, respectively *p* < 0.0001), higher eGFR (49.7 ± 14.7, 55.1 ± 19.7, 62.5 ± 21.6 and 64.4 ± 23.4 in groups 1–4, respectively *p* < 0.0001), reduction in the pre-procedural use of aspirin (68%, 68%, 58% and 56% in groups 1–4, respectively *p* < 0.0001) and angiotensin-converting enzyme inhibitors (59%, 59%, 52% and 22% in groups 1–4, respectively *p* = 0.02).

Echocardiographic baseline parameters, including valve estimation, did not differ significantly between the different groups.

### 3.2. Procedural Outcomes

When analyzing the procedural outcomes (Table 2), a significant trend was seen in the use of balloon pre-dilatation (99%, 72%, 26% and 18% in groups 1–4, respectively. *p* < 0.0001). The use of balloon post-dilatation peaked in group 3 (2016–2018) but still presented a significant trend for value (0.9%, 7.1%, 29% and 14% in groups 1–4, respectively *p* < 0.0001). Additional improved outcomes included reduced perivalvular leak (estimated by angiography) (2.8%, 0.8%, 0.7% and 0% in groups 1–4, respectively *p* < 0.0001) and valve success as measured by VARC2 (96.3%, 95.2%, 98.1% and 99.2% in groups 1–4, respectively *p* < 0.0001).

### 3.3. Hospitalization Features

**Figure 1:**
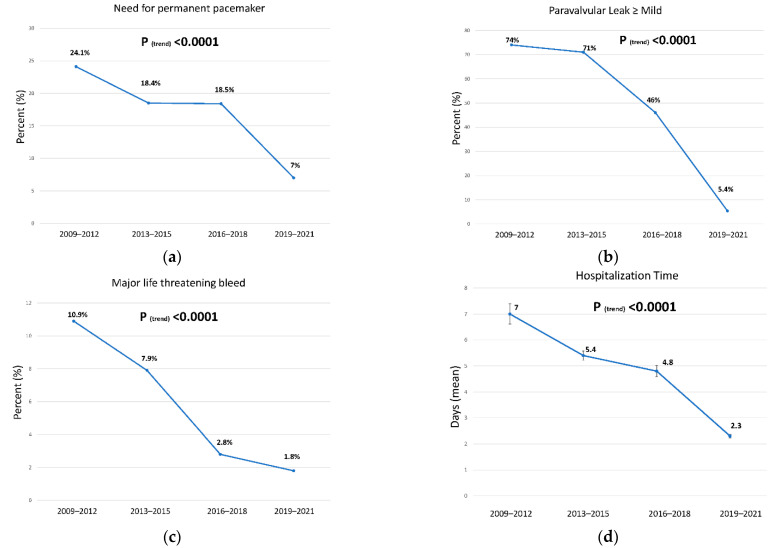
Major results of the cohort. (**a**). The need for permanent pacemaker placement after the procedure (**b**). Rate in percent of paravalvular leak above mild as measured by echocardiography after the procedure (**c**). Rate (in percent) of life-threatening bleed (**d**). Mean hospitalization time.

**Figure 2:**
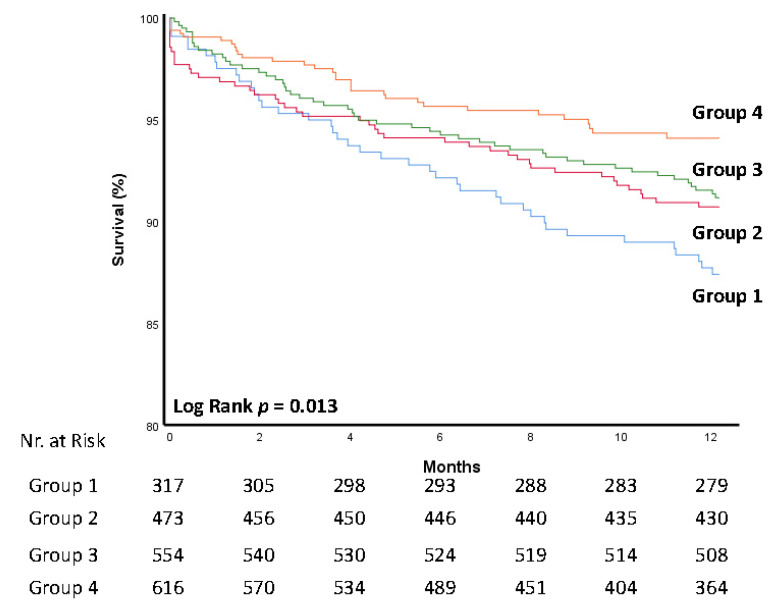
Kaplan–Meier curve of mortality rates in the different study groups. For 1-year survival rates, group were divided by procedure date: 2009–2102, 2013–2015, 2016–2018 and 2019–2021 for groups 1–4, respectively.

Post-procedural hospitalization analysis (Table 3 and Figure 1) revealed a lower complication rate for conduction abnormalities, including left and right bundle branch block or a need for a permanent pacemaker (24.1%, 18.5%, 18.4% and 7% in groups 1–4, respectively *p* < 0.0001). Post-procedural bleeding was lower over the years (10.9%, 7.9%, 2.8% and 1.8% in groups 1–4, respectively. *p* < 0.0001), and although higher volumes of contrast were used, a lower acute kidney injury rate was noted (13.7%, 16%, 5.1% and 1.1% in groups 1–4, respectively *p* < 0.0001). Hospital admission time was also reduced from 7 ± 7.1 days in group 1 to 2.3 ± 1.7 in group 4 (*p* < 0.0001). Finally, post-procedural echocardiography demonstrated reduced rates of paravalvular leak (*p* < 0.0001). Mortality rates were significantly lower at 30 days (*p* < 0.0001) and at 1 year (*p* = 0.013) (Figure 2). Multivariate analysis performed found that TAVR date was an independent protector from all-cause mortality when calculated per group as well as per year (HR 0.847 CI (0.76–0.94) *p* value 0.002 and HR 0.95 CI (0.92–0.98) *p* value 0.004, respectively). Other independent factors are described in Table 4.

## 4. Discussion

Our study depicted the trends in TAVR procedure over a 12-year period, as experienced by a tertiary large-scale referral center. We demonstrated significant trends in several aspects of the TAVR procedure, starting from patient characteristics through the procedural outcomes with improved device success and lower rates of complications and finally in reduced mortality and shortened hospital stay.

As mentioned previously, TAVR, a procedure that was once reserved for the high-surgical-risk population, is now being performed in a wider variety of clinical settings due to its improved safety profile [1,2,4]. Considering these changes, patients’ characteristics are changing as well; younger patients with lower surgical risk scores (Euroscore2 was 4.6 in group 1 vs. 3.1 in group 4) are now being accepted. This change is largely attributed to the expansion of indications and better patient selection, as seen in previous studies [15,16,17], as well as changing guidelines—which now partially recommend TAVR for intermediate surgical risk patients [6]. It is worth mentioning that the aortic disease properties did not differ over the years, as seen by ejection fraction and valve measurements, which heightens the notion that the pure indication for intervention (the definition of severe AS) has not been modified over the years [5,6].

The procedure itself also transformed during our study period, with growing operator experience and confidence. Previous studies have demonstrated that an experienced physician reduces the rate of procedural complications [18]. Our cohort, comprising a single center with highly experienced operators, has shown a reduced use of balloon pre-dilation (99% to 18%). Similar rates were described in previous works such as the DIRECT trial [19]. The reduction in pre-balloon dilatation did not result in a rise in device failure or moderate paravalvular leak (as per angiogram). On the contrary, device success, defined by VARC2, rose from 96% to 99.2%, and moderate paravalvular leak as per an angiogram was reduced from 9% to 0%.

The TAVR procedure encompasses a known array of complications, such as stroke, conduction abnormalities, vascular access and bleeding [8,9]. Conduction abnormalities are the most known and studied complications. Numerous studies have tried to assess the risk for persistent heart block and especially the need for permanent pacemaker (PPM) implantation, yet their results are inconsistent. An example is a systemic review of 41 studies, which reported a 0.5% to 50% prevalence of PPM [20]. When trying to evaluate a trend over several years, studies found no change in PPM placement [21]. One study even described a rise in high-degree AV block (9.5% to 13.7% during the years 2012–2015) [22]. Our results show a reduced rate of all clinically significant heart blocks and in the need for PPM (24% to 7%). This reduction is attributed mainly to the new perception that conduction abnormalities can be transient and watchful waiting may be in order for most cases, as described in ESC guidelines [5].

Post-procedural bleeding is a particularly important complication, as previous studies linked it to increased 30-day mortality and determined it to be a contributing factor for acute kidney injury [23,24]. The risk for early bleeding stems from many factors, including puncture site injury, advanced age, post-procedural thrombocytopenia and von Willebrand deficiency [25]. We demonstrated a negative trend in this known complication that is attributed to a global trend of reduction in sheath size for vascular access [26] as well as to younger generally healthier patients with less comorbidities, as discussed earlier.

Finally, two additional significant trends were seen in our analysis—mortality reduction and shortened hospitalization time. It is hard to overestimate the value of reduced hospital stay in terms of patient wellbeing, a reduced rate of hospital-related complications and admission cost. Our results lay in accordance with previous studies that showed reduced post-procedural hospital stay [21,27,28,29]. In our cohort, hospitalization stay was 7 ± 7.1 in group 1, compared with 2.3 ± 1.7 in group 4. This significant reduction in hospital stay further enhances the relative advantage of TAVR over surgical valve replacement. Mortality rates were significantly lower at 30 days (*p* < 0.0001) and at 1 year (*p* = 0.013). Reduced mortality rates can be attributed to improved operator techniques, reduction in the rates of perioperative complications and changing patient profiles, as discussed above. It is worth mentioning that a later procedure date was found to be an independent protector from all-cause mortality, a fact the enhances the protective role of operator skills and equipment. These results suggest that TAVR, while still a procedure offered mainly to high-risk patients with significant morbidity and mortality, has become relatively safer.

Our study has several limitations. Most importantly, this is a single-center experience, and therefore its generalizability to the wide population is limited. In addition, a single-center experience also represents a limited number of operating physicians, a fact that can create some bias. Furthermore, data were collected retrospectively, and no follow-up visits were included in the present analysis in order to assess patient symptoms, quality of life and valve durability. Being a tertiary care center, a referral bias could not be excluded.

In conclusion, this study described the trends in a relatively large TAVR population in a high-volume medical center. Our results show that patients selected for this procedure were at a lower surgical risk over the years, as expected by changing guidelines, and that TAVR outcomes were improved with fewer known complications, reduced mortality rate and shortened hospital stay. These encouraging results are attributed mainly to operator experience as well as changes in practice and guidelines. Future similar studies may establish the procedural trends and enable clinicians to offer TAVR to a more diverse population.

## Figures and Tables

**Table 1:** Baseline Characteristics. ACEI—angiotensin-converting enzyme inhibitor, ARB—angiotensin receptor blocker, CABG—coronary artery bypass graft. eGFR—estimated glomerular filtration rate, ICD—implantable cardioverter defibrillator, IQR—interquartile range LAHB—Left anterior hemiblock, MDRD—modification of diet in renal disease, NYHA—New York Heart Association, RBBB-Right bundle branch block, SD—standard deviation, TIA—transient ischemic attack.

1988	Group 1 (2009–2012) *n* = 321	Group 2 (2013–2015) *n* = 482	Group 3 (2016–2018) *n* = 565	Group 4 (2019–2021) *n* = 620	*p* Value (for Trend)
Age (years), mean ± SD	84.4 ± 5.6	83.7 ± 6.2	82.1 ± 6.3	80.3 ± 6.6	<0.0001
Gender (female), *n (*%)	193 (60)	253 (53)	294 (52)	272 (44)	<0.0001
Body mass index (kg/m^2^), median (IQR)	26.6 (23.9–29.8)	26.2 (23.5–29.6)	26.6 (24.1–29.7)	26.9 (24.1–30.1)	0.19
Body surface area(m^2^), mean ± SD	1.79 ± 0.21	1.80 ± 0.19	1.81 ± 0.21	1.85 ± 0.21	<0.0001
EuroSCORE 2, median (IQR)	4.6 (3.0–7.3)	4.3 (2.6–7.4)	3.4 (2.2–5.6)	2.8 (1.8–5.1)	<0.0001
NYHA functional class > 2	312 (97)	412 (86)	480 (85)	458 (74)	<0.0001
Prior stroke/TIA, *n (*%)	34 (11)	73 (15)	75 (13)	63 (10)	0.31
Diabetes mellitus, *n (*%)	104 (32)	191 (40)	203 (36)	234 (38)	0.34
Hypertension, *n (*%)	282 (88)	420 (87)	470 (84)	500 (81)	0.001
Frailty, *n (*%)	43 (13)	116 (24)	178 (31)	148 (24)	0.001
Chronic obstructive pulmonary disease, *n (*%)	63 (20)	56 (12)	53 (9)	51 (8)	<0.0001
Chronic dialysis, *n (*%)	5 (1.6)	10 (2.1)	15 (2.7)	14 (2.3)	0.46
Atrial fibrillation/flutter, *n (*%)	97 (30)	158 (33)	146 (26)	172 (28)	0.11
Coronary artery disease, *n (*%)	184 (57)	299 (62)	241 (43)	290 (47)	<0.0001
Prior myocardial infarction, *n (*%)	51 (16)	96 (20)	53 (9)	17 (5)	<0.0001
Prior CABG *n (*%)	51 (16)	77 (16)	79 (14)	80 (24)	-
Prior pacemaker/ICD, *n (*%)	27 (8)	66 (14)	93 (17)	88 (14)	0.02
Prior valve intervention (non aortic valve), *n (*%)	2 (0.6)	11 (2.3)	6 (1.1)	15 (2.4)	0.16
Hemoglobin (g/dL), mean ± SD	11.9 ± 1.4	11.9 ± 1.5	12.1 ± 1.6	12.6 ± 1.6	<0.0001
Creatinine (mg/dL), median (IQR)	1.2 (1.0–1.4)	1.1 (0.9–1.4)	1.0 (0.8–1.3)	0.9 (0.8–1.3)	<0.0001
eGFR (MDRD formula, mL/min/1.73^2^), mean ± SD	49.7 ± 14.7	55.1 ± 19.7	62.5 ± 21.6	64.4 ± 23.4	<0.0001
Aspirin	219 (68)	329 (68)	330 (58)	350 (56)	<0.0001
Beta blockers	170 (53)	290 (60)	314 (55)	331 (53)	0.42
ACEI/ARB	188 (59)	284 (59)	235 (42)	139 (22)	<0.0001
Statins	217 (68)	346 (71)	387 (69)	388 (63)	0.02
Baseline RBBB, *n (*%)	Missing	30 (6.2)	66 (11.7)	59 (9.5)	0.09
Baseline LAHB, *n (*%)	Missing	36 (7.5)	32 (5.7)	36 (5.8)	0.28
Baseline RBBB and LAHB, *n (*%)	Missing	10 (2.1)	16 (2.8)	14 (2.3)	0.89
Baseline echocardiographic parameters					
Ejection fraction ≤ 45%, *n (*%) (Semiquantitative)	24 (7.5)	50 (10.4)	54 (9.6)	49 (7.9)	0.76
Aortic valve peak pressure (mmHg), mean ± SD	78.1 ± 22.8	74.6 ± 22.5	74.8 ± 22.1	77.8 ± 20.2	0.87
Aortic valve mean pressure (mmHg), mean ± SD	47.5 ± 14.7	45.8 ± 14.7	45.2 ± 14.4	47.7 ± 13.7	0.98
Aortic valve area (cm^2^), mean ± SD	0.69 ± 0.18	0.74 ± 0.18	0.75 ± 0.16	0.72 ± 0.19	0.11
Systolic pulmonary artery pressure (mmHg), median (IQR)	39 (30–50)	38 (30–50)	37 (31–49)	37 (30–49)	0.71

**Table 2:** Procedural Features. CPR—cardiopulmonary resuscitation, IQR—interquartile range, LVOT—left ventricular outlet tract, SD—standard deviation, VARC—Valve Academic Research Consortium, VT—ventricular tachycardia, VF—ventricular fibrillation.

*n* = 1988	Group 1 (2009–2012) *n* = 321	Group 2 (2013–2015) *n* = 482	Group 3 (2016–2018) *n* = 565	Group 4 (2019–2021) *n* = 620	*p* Value (for Trend)
Balloon pre-dilatation, *n (*%)	317 (99)	345 (72)	148 (26)	113 (18)	<0.0001
Balloon post-dilatation, *n (*%)	3 (0.9)	34 (7.1)	164 (29)	88 (14)	<0.0001
Contrast volume (mL), median (IQR)	140 (115–168)	150 (121–165)	162 (140–197)	156 (135–186)	<0.0001
Fluoroscopy time (min), mean ± SD	16.4 ± 5.9	16.1 ± 5.5	15.1 ± 6.9	15.2 ± 6.9	0.01
Valve type					<0.0001
Edwards lifesciences	65 (20)	191 (40)	246 (44)	269 (48)	
Medtronic	254 (80)	274 (57)	314 (55.5)	254 (45)	
Other	0 (0)	15 (3)	3 (0.5)	37 (7)	
Valve size	26.7 ± 1.9	26.6 ± 2.1	27.1 ± 2.5	27.2 ± 3.0	<0.0001
Device success (VARC2), *n (*%)	309 (96.3)	459 (95.2)	554 (98.1)	615 (99.2)	<0.0001
Perivalvular leak ≥ moderate (per angio)	9 (2.8)	4 (0.8)	4 (0.7)	0 (0)	<0.0001
Need for 2nd valve, *n (*%)	3 (0.9)	10 (2.1)	6 (1.1)	9 (1.5)	0.98
Conversion to open surgery, *n (*%)	1 (0.3)	2 (0.4)	3 (0.5)	1 (0.2)	0.66
Need for cardiopulmonary bypass, *n (*%)	0 (0)	0 (0)	1 (0.2)	1 (0.2)	0.32
Coronary obstruction, *n (*%)	1 (0.3)	1 (0.2)	0 (0)	2 (0.3)	0.99
Ventricular septal perforation, *n (*%)	0 (0)	0 (0)	0 (0)	0 (0)	-
Mitral valve damage, *n (*%)	1 (0.3)	1 (0.2)	0 (0)	0 (0)	0.09
Tamponade, *n (*%)	3 (0.9)	8 (1.7)	4 (0.7)	3 (0.5)	0.15
Annular rupture, *n (*%)	0 (0)	3 (0.6)	0 (0)	0 (0)	0.22
Valve malpositioning, *n (*%)	0 (0)	0 (0)	3 (0.5)	0 (0)	0.68
Valve migration or embolization, *n (*%)	4 (1.2)	5 (1.0)	0 (0)	5 (0.8)	0.26
Procedural CPR, *n (*%)	0 (0)	1 (0.2)	1 (0.2)	4 (0.6)	0.08
Procedural VT/VF(requiring treatment), *n (*%)	1 (0.3)	4 (0.8)	3 (0.5)	5 (0.8)	0.55
LVOT obstruction, *n (*%)	0 (0)	0 (0)	0 (0)	0 (0)	-

**Table 3:** Hospitalization features. AVB—atrioventricular block, CAVB—complete atrioventricular block CVA—cerebrovascular accident, IQR—interquartile range, LBBB—left bundle branch block, MI—myocardial infraction, PPM—permanent pacemaker, RBBB—right bundle branch block, SD—standard deviation, TIA—transient ischemic attack.

*n* = 1988	Group 1 (2009–2012) *n* = 321	Group 2 (2013–2015) *n* = 482	Group 3 (2016–2018) *n* = 565	Group 4 (2019–2021) *n* = 620	*p* Value (for Trend)
New LBBB, *n (*%) (only patients with no prior LBBB, *n* = 1867)	64 (19)	100 (22)	143 (28)	89 (15)	0.12
New RBBB, *n (*%) (only patients with no prior RBBB, *n* = 1833)	12 (3.7)	10 (2.2)	10 (2.0)	3 (0.5)	0.001
New AVB ≥ 2 degree, *n (*%) (only patients with no prior pacemaker, *n* = 1714)	44 (15.0)	47 (11.3)	40 (8.5)	36 (6.8)	<0.0001
New CAVB, *n (*%) (only patients with no prior pacemaker, *n* = 1714)	38 (12.9)	39 (9.4)	35 (7.4)	32 (6.0)	<0.0001
New need for PPM, *n (*%) (only patients with no prior pacemaker, *n* = 1714)	71 (24.1)	77 (18.5)	87 (18.4)	37 (7.0)	<0.0001
Major or life-threatening bleeding, *n (*%)	35 (10.9)	38 (7.9)	16 (2.8)	11 (1.8)	<0.0001
Major vascular complications, *n (*%)	28 (8.7)	31 (6.4)	6 (1.1)	44 (7.1)	0.16
Acute kidney injury ≥ Stage 1, *n (*%)	44 (13.7)	77 (16.0)	29 (5.1)	7 (1.1)	<0.0001
Acute kidney injury ≥ Stage 2, *n (*%)	4 (1.2)	9 (1.9)	9 (1.6)	0 (0)	0.02
Acute dialysis, *n (*%) (only patients without chronic dialysis, *n* = 1944)	1 (0.3)	0 (0)	1 (0.2)	2 (0.3)	0.63
Periprocedural MI ( <72 h), *n (*%)	0 (0)	0 (0)	1 (0.2)	0 (0)	0.81
Spontaneous MI ( >72 h), *n (*%)	0 (0)	0 (0)	0 (0)	0 (0)	-
Periprocedural CVA/TIA, *n (*%)	5 (1.6)	5 (1.0)	13 (2.3)	3 (0.5)	0.32
Days until discharge (days), mean ± SD	7.0 ± 7.1	5.4 ± 3.9	4.8 ± 5.3	2.3 ± 1.7	<0.0001
Post-procedural echocardiography					
Ejection fraction ≤ 45%, *n (*%) (*n* = 1576)	8 (6.3)	24 (6.3)	28 (5.3)	33 (6.1)	0.88
Ejection fraction (%), mean ± SD, (*n* = 1091)	57.1 ± 6.7	56.5 ± 6.8	56.9 ± 6.9	56.4 ± 6.9	0.66
Aortic stenosis ≥ mild, *n (*%), (*n* = 703)	23 (79)	102 (75)	33 (61)	4 (0.8)	<0.0001
Aortic stenosis ≥ moderate, *n (*%), (*n* = 703)	3 (10.3)	4 (2.9)	1 (1.9)	1 (0.2)	<0.0001
Paravalvular leak ≥ mild, *n (*%), (*n* = 1467)	224 (74)	322 (71)	93 (46)	27 (5.4)	<0.0001
Paravalvular leak ≥ mild-to-moderate, *n (*%), (*n* = 1467)	9 (3)	38 (8.3)	18 (8.8)	15 (3.0)	0.29
Aortic valve peak pressure (mmHg), mean ± SD, (*n* = 1553)	17.8 ± 9.3	18.4 ± 10.1	17.9 ± 10.1	19.1 ± 8.5	0.21
Aortic valve mean pressure (mmHg), mean ± SD, (*n* = 1508)	9.8 ± 5.3	10.3 ± 5.9	10.2 ± 6.2	10.7 ± 4.6	0.13
Aortic valve area cm (mean ± SD), (*n* = 324)	-	-	1.86 ± 0.49	1.78 ± 0.45	-
Systolic pulmonary artery pressure (mmHg), median (IQR)	39 (31–46)	39 (31–49)	37 (31–47)	37 (31–46)	0.31
Procedural mortality, *n (*%)	0 (0)	6 (1.2)	1 (0.2)	3 (0.5)	0.89
In-hospital mortality, *n (*%)	5 (1.6)	4 (0.8)	6 (1.1)	1 (0.2)	0.03

**Table 4:** Univariate and multivariate regressions. Univariate regression was performed for all significant baseline variables. Only significant predictors are presented. Multivariate regression was performed in two models—the first for TAVR by procedure year and the second for TAVR by our divided groups. Abbreviations: BMI—body mass index, NYHA—New York Heart Association, CVA—cerebrovascular accident, COPD—chronic obstructive pulmonary disease, ICD—implantable cardioverter-defibrillator, LVEF—left ventricular ejection fraction, TAVR—trans-aortic valve repair.

Variable	Univariate HR (CI) *p* Value	Multivariate Model 2 HR (CI) *p* Value	Multivariate Model 1 HR (CI) *p* Value
TAVR (years)	0.94 (0.92–0.97) <0.001	--------	0.95 (0.92–0.98) 0.004
TAVR (groups)	0.84 (0.77–0.92) <0.001	0.85 (0.76–0.94) 0.002	--------
Male Gender	1.21 (1.05–1.39) 0.008	1.29 (1.09–1.52) 0.002	1.28 (1.09–1.51) 0.003
Age	1.04 (1.03–1.05) <0.001	1.02 (1.01–1.04) 0.001	1.02 (1.01–1.04) <0.001
BMI (kg/m)	0.98 (0.97–0.99) 0.03	0.99 (0.98–1.02) 0.833	0.99 (0.98–1.015) 0.84
EuroSCORE 2	1.04 (1.03–1.05) <0.001	0.99 (0.98–1.01) 0.602	0.99 (0.98–1.01) 0.63
NYHA class	2.15 (1.57–2.94) <0.001	1.76 (1.26–2.46) 0.001	1.76 (1.26–2.47) 0.001
Prior CVA	1.31 (1.07–1.59) 0.008	1.1 (0.89–1.36) 0.359	1.11 (0.9–1.37) 0.32
Diabetes mellitus	1.20 (1.04–1.39) 0.01	1.16 (0.99–1.36) 0.62	1.17 (0.99–1.37) 0.59
Frailty	1.52 (1.29–1.78) <0.001	1.32 (1.1–1.58) 0.002	1.31 (1.09–1.56) 0.003
COPD	1.54 (1.27–1.86) <0.001	1	1.46 (1.19–1.76) <0.001
Chronic dialysis	2.67 (1.77–4.01) <0.001	2.06 (1.32–3.24) 0.002	2.09 (1.33–3.27) 0.001
Atrial fibrillation or flutter	1.62 (1.39–1.88) <0.001	1.41 (1.2–1.65) <0.001	1.41 (1.2–1.65) <0.001
Coronary artery disease	1.28(1.11–1.47) 0.001	1.06 (0.89–1.25) 0.48	1.06 (0.89–1.25) 0.48
Prior myocardial infraction	1.62(1.34–1.94) <0.001	1.18 (0.96–1.47) 0.11	1.18 (0.96–1.47) 0.11
Pacemaker/ICD	1.62(1.34–1.94) <0.001	1.28 (1.03–1.59) 0.26	1.27 (1.02–1.58) 0.31
Left bundle branch block	0.77(0.64–0.93) 0.007	1.02 (0.82–1.27) 0.82	0.99 (0.8–1.22) 0.95
Baseline LVEF < 45%	1.49(1.19–1.87) <0.001	0.96 (0.73–1.3) 0.77	0.96 (0.73–1.26) 0.79

## Data Availability

The data presented in this study are available on request from the corresponding author.
